# Cardiac Vagal Decoupling in Canine Anesthesia: A Physiological Concept for Graded Vasopressor Titration

**DOI:** 10.3390/vetsci13101121

**Published:** 2026-10-21

**Authors:** Shotaro Nagahama

**Affiliations:** 1 JAVA Incorporated Association, Tokyo 108-0075, Japan; nagahama.s@java2016.com; 2 Department of Veterinary Anesthesia, Veterinary Teaching Hospital, Faculty of Applied Biological Sciences, Gifu University, Gifu 501-1193, Japan; 3 Laboratory of Veterinary Surgery, Graduate School of Agricultural and Life Sciences, The University of Tokyo, Tokyo 113-8657, Japan

**Keywords:** atropine, baroreflex, cardiac vagal decoupling, dog, general anesthesia, intraoperative hypotension, reflex bradycardia, vasopressor titration

## Abstract

During general anesthesia in dogs, vasopressor-induced increases in mean arterial pressure (MAP) may be partly opposed by reflex sinus slowing. Subsequent administration of atropine or another antimuscarinic may cause an abrupt increase in sinus rate; when vascular tone is already elevated, this increase may in turn contribute to an abrupt or excessive MAP rise. This article examines cardiac vagal decoupling, defined as reduced vagally mediated muscarinic restraint at the sinoatrial node, as a physiological concept for graded vasopressor titration. The hypothesis is that reducing this restraint before substantial vasopressor escalation, followed by MAP-guided dose adjustments, may permit more graded pressure control: reaching and maintaining a specified MAP range with fewer abrupt or excessive pressure increases. Canine studies support muscarinic control of sinus rate, baroreflex responses during anesthesia, and modification of vasopressor responses by prior antimuscarinic administration. These findings support components of the proposed concept, but whether it improves graded MAP titration has not been directly tested. The article contrasts the two hemodynamic patterns, evaluates the supporting evidence, and considers the limits of the hypothesis. Effects of such titration on cardiac output, tissue perfusion, and clinical outcomes require separate evaluation.

## Simple Summary

Blood pressure can fall during general anesthesia in dogs. Drugs used to raise it may also trigger a reflex that slows the heart, partly limiting the rise in pressure. Atropine given afterward can reduce this slowing effect and may cause heart rate to rise abruptly. If blood vessels are already constricted, that rise in heart rate may in turn contribute to an abrupt increase in blood pressure. This article considers whether reducing the vagus nerve’s slowing effect on the heart before increasing drugs used to raise blood pressure could allow subsequent blood pressure adjustment in smaller steps. The proposal is called cardiac vagal decoupling and assumes that these drugs are adjusted according to the observed blood pressure response. Published studies support individual components of this idea, but whether it improves blood pressure adjustment remains untested. The article therefore presents a hypothesis for investigation rather than a treatment protocol. Effects on blood flow to organs and patient outcomes would require separate evaluation.

## 1. Introduction

Intraoperative hypotension in dogs is commonly managed by adjusting anesthetic drug administration, fluid therapy, and vasoactive support according to the suspected cause. In many cases, mean arterial pressure (MAP) is the main bedside target. The problem considered here arises when a vasopressor-associated MAP increase is accompanied by slowing of heart rate (HR; more precisely, sinus rate when sinus rhythm is present). A similar MAP increase can occur with sinus-rate slowing or preserved sinus rate, while the resulting cardiac output depends on the accompanying stroke-volume response.

Cardiac vagal decoupling denotes reduced vagally mediated muscarinic restraint at the sinoatrial node. The term identifies a cardiac effector action, not interruption of pressure sensing, central reflex integration, or the sympathetic limb of the baroreflex. Our prior human-anesthesia paper introduced this concept and hypothesized that reducing vagal restraint before substantial vasopressor escalation might allow more proportional vasopressor responses and reduce hypertensive overshoot compared with later antimuscarinic administration [1]. Drawing on canine physiological and clinical evidence, this article further develops the hypothesis that prior reduction of sinoatrial muscarinic restraint may facilitate graded blood pressure control during MAP-guided vasopressor titration.

The article focuses on this sequence-dependent component of the vasopressor response rather than global cardiovascular performance.

## 2. Two Hemodynamic Patterns During Pressor Titration

**Figure 1:**
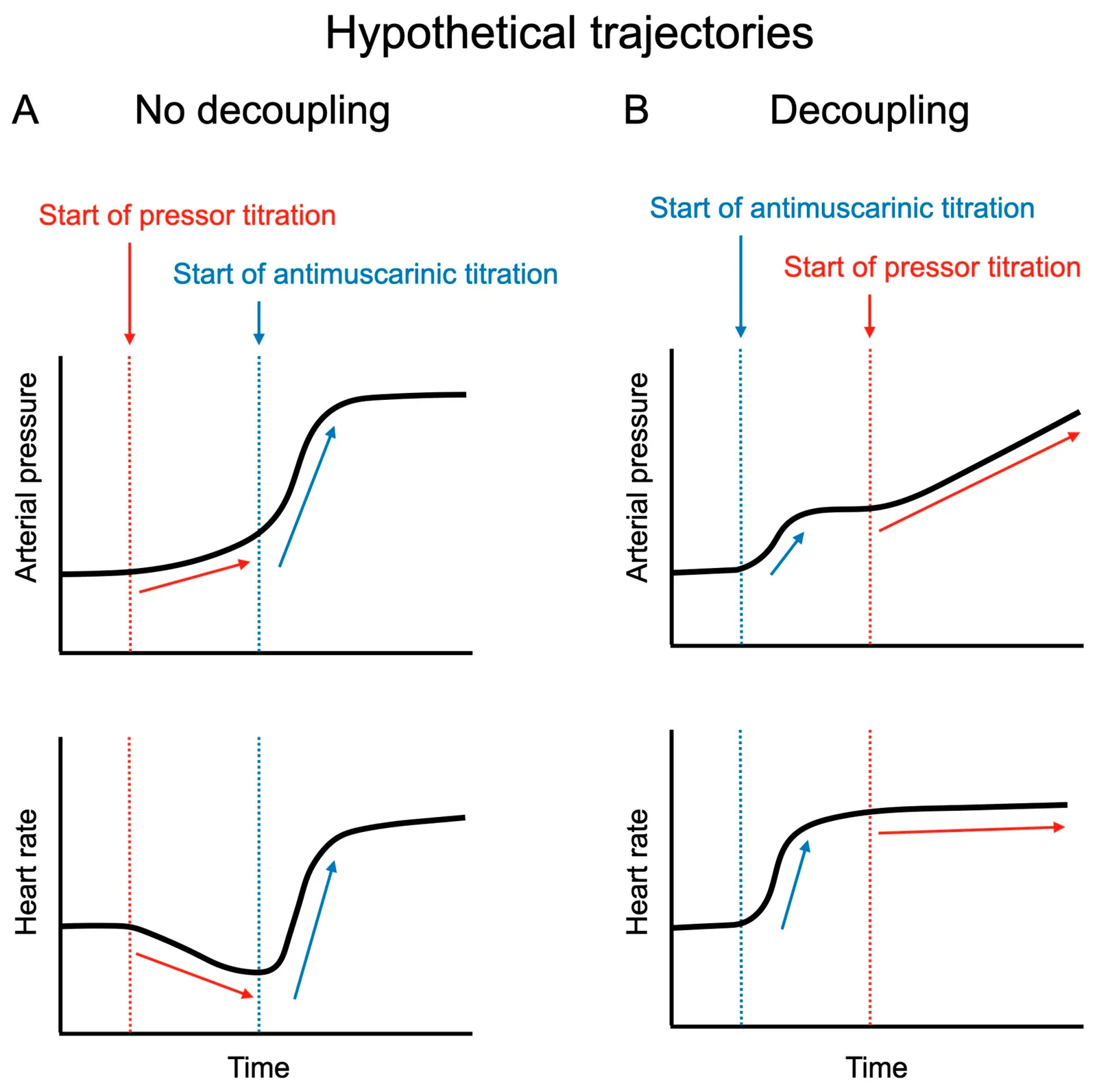
**Hypothetical hemodynamic trajectories in dogs without versus with cardiac vagal decoupling (schematic; not experimentally observed; not to scale).** (**A**) **No decoupling.** At the start of pressor titration, arterial pressure increases while reflex-linked sinoatrial muscarinic restraint slows sinus rate, partly opposing the MAP rise. When the antimuscarinic effect is introduced later, sinus rate may increase abruptly. On an already elevated vascular-tone background, this rate increase may in turn contribute to an abrupt MAP rise beyond the intended range. The steep sinus-rate change illustrates the proposed non-proportional expression of the antimuscarinic effect; the vascular-tone background may amplify its pressure consequence. (**B**) **Decoupling.** Functional muscarinic restraint at the sinoatrial node is reduced before substantial pressor escalation. Subsequent pressor titration is then predicted to be less consistently constrained by reflex sinus slowing. The illustrated trajectory represents the hypothesized response to small vasopressor adjustments guided by the observed MAP. Neither panel depicts an experimentally demonstrated trajectory. The curves illustrate possible relative changes, and the smoother MAP rise in panel B is the prediction to be tested, not an established effect. HR responses may vary with baseline autonomic activity, anesthetic drug effects, cardiovascular condition, and the agents administered; the near-flat segment in panel B does not imply a constant HR. ‘Pressor titration’ denotes MAP-guided vasopressor dose adjustment; drug class is a modifier of the hypothesized response (Section 4). ‘Antimuscarinic titration’ in the figure denotes the introduction or adjustment of the antimuscarinic effect at the sinoatrial node. ‘Decoupling’ denotes reduced functional muscarinic restraint before substantial pressor escalation; it does not specify a drug, dose, route, or mandatory clinical sequence.

Figure 1 contrasts two hypothetical response patterns. In the non-decoupled pattern, pressor titration begins while functional sinoatrial muscarinic restraint remains influential. In the decoupled pattern, pressor titration begins after that restraint has already been reduced. In Figure 1A, as MAP rises, reflex-linked vagal influence can slow sinus rate, so the intended MAP increase may be partly opposed by reflex sinus slowing.

The latter part of Figure 1A illustrates one possible mechanism by which a late antimuscarinic effect could contribute to an abrupt MAP rise. Atropine or another antimuscarinic may cause sinus rate to rise abruptly. If vascular tone is already high, that rate increase may in turn contribute to an abrupt or excessive increase in MAP. The magnitude of the pressure change also depends on venous return, ventricular filling, stroke volume, and vascular resistance, rather than following the rate change proportionally. Incremental drug administration does not necessarily produce proportional sinus-rate increments, as discussed in Section 5.

Figure 1B illustrates the decoupled pattern. Functional muscarinic restraint at the sinoatrial node is reduced before substantial vasopressor escalation. Subsequent vasopressor titration is expected to be less constrained by reflex sinus slowing. The hypothesis is that small vasopressor dose adjustments guided by the observed MAP response may permit more graded pressure control.

Operationally, the relevant physiological change is less sinus-rate slowing for a comparable pressor-induced pressure rise, assessed separately from the success of MAP control. Neither the HR attained after atropine nor its absolute or percentage increase from baseline can, by itself, quantify the degree of cardiac vagal decoupling. It neither requires a target HR nor implies complete vagolysis.

## 3. Why Dogs Are a Relevant Setting

Dogs are a clinically important setting for this concept because available evidence suggests substantial vagally mediated muscarinic influence at the sinoatrial node [2,3].

In conscious dogs, Little et al. found that cumulative atropine administration shortened mean R–R intervals and reduced a real-time index of cardiac vagal activity derived from those intervals to near zero [2]. O’Leary et al. reported that atropine markedly increased HR relative to saline treatment during treadmill exercise in conscious dogs, with little effect on MAP even at high workloads [3]. These studies do not reproduce the anesthetized clinical setting, but they establish that the canine sinus node can operate under substantial and dynamically modifiable vagal restraint.

Schuessler et al. varied arterial pressure with phenylephrine and nitroprusside in chloralose-morphine-anesthetized, open-chest dogs and recorded changes in HR and the sites initiating atrial activation. Experiments with atropine and propranolol supported autonomic mediation of these baroreflex HR responses [4]. Tanaka and Nishikawa assessed baroreflex sensitivity in pentobarbital-anesthetized dogs by measuring the relationship between MAP and R–R intervals during phenylephrine-induced pressure increases and nitroprusside-induced decreases. Baroreflex sensitivity did not change significantly after acute normovolemic hemodilution [5]. Frankel et al. calculated spontaneous baroreflex sensitivity from the regression of beat-to-beat changes in pulse interval on changes in systolic pressure in conscious dogs. This measure was abolished by atropine, ganglionic blockade with hexamethonium, and bilateral carotid sinus denervation [6].

Fahim and Arndt compared baroreflex HR responses in anesthetized, mechanically ventilated, open-chest dogs and cats and found greater sensitivity in dogs [7]. The chronotropic responses involved both sympathetic and parasympathetic pathways, so these findings do not establish a specifically vagal basis for the species difference.

Seagard et al. examined baroreflex HR responses at different isoflurane concentrations. When anesthetic-induced changes in blood pressure were prevented, significant depression of the baroreflex HR response occurred only at the higher tested concentration [8]. Bagshaw and Cox measured central and regional hemodynamics while varying carotid sinus pressure and isoflurane concentration. Isoflurane reduced the mean aortic pressure response to carotid sinus stimulation at the reflex set point [9]. Szilagyi used phenylephrine and nitroprusside to assess baroreflex HR responses in anesthetized dogs and found that naloxone reduced baroreflex sensitivity [10]. Together, these studies support muscarinic modulation of cardiac rate, reflex cardiac responses to pressure changes, and modification of those responses by autonomic and anesthetic conditions. They establish the physiological substrate in dogs, rather than improved MAP control during repeated vasopressor dose adjustment.

## 4. Experimental and Clinical Evidence Supporting the Concept

The two Seki et al. studies examined cardiovascular responses to ephedrine with and without prior atropine. In a single-blind crossover experiment in six healthy Beagles with isoflurane-induced hypotension, each dog received a fixed intravenous ephedrine bolus after atropine or saline pretreatment [11]. Stroke volume and cardiac output were estimated by Doppler echocardiography and reported as indexed values. Prior atropine prevented the ephedrine-associated HR decrease and was associated with a greater early MAP rise and higher post-ephedrine cardiac index values than saline pretreatment. Baseline measurements were obtained 15 min after atropine or saline, immediately before ephedrine administration. Organ blood flow and tissue perfusion were not measured.

In a single-center retrospective analysis of anesthetized dogs and cats receiving an initial ephedrine bolus, preanesthetic atropine was associated with less frequent HR decreases of ≥20% and a higher rate of reaching the study’s response criterion (MAP > 70 mmHg after ephedrine) in dogs [12]. In cats, atropine premedication was not significantly associated with attainment of this MAP criterion. These associations do not establish causation.

Alibhai et al. examined the cardiovascular effects of intramuscular medetomidine with and without atropine in six dogs [13]. When atropine was administered immediately after medetomidine, initial bradycardia was followed by a rapid HR increase 10–15 min after injection and marked hypertension. Marked hypertension also occurred when atropine preceded medetomidine by 30 min; HR fell after medetomidine but remained higher than with medetomidine alone. Intermittent tachyarrhythmias were observed with both combinations. The authors interpreted the hypertension as resulting from medetomidine-induced vasoconstriction in the presence of higher HR. Medetomidine was administered at a fixed dose rather than adjusted to the observed MAP response.

A greater MAP response to a fixed dose is distinct from easier control of MAP during successive dose adjustments. The titration hypothesis concerns whether reducing sinoatrial muscarinic restraint, followed by vasopressor adjustments guided by the observed MAP response, permits a more graded approach to the intended pressure. This question was not tested by the cited studies.

Vasopressor class may modify the proposed response. Ephedrine has direct α- and β-adrenergic actions and indirect effects through endogenous norepinephrine release [14]. Its interaction with antimuscarinic pretreatment therefore cannot establish an identical response to predominantly vasoconstrictor agents. The hypothesis requires drug-specific testing rather than assuming a uniform effect across vasopressors.

## 5. Antimuscarinic Effect Expression and Graded Titration

The non-decoupled pattern raises a practical question: if a late antimuscarinic effect can produce an abrupt MAP-HR response, why not simply titrate the antimuscarinic drug more gradually? The key distinction is between titrating drug exposure and titrating the observable sinus-rate response. Drug exposure can be introduced incrementally, but a proportional sinus-rate response cannot be assumed.

In seven dogs with morphine- and fentanyl-induced bradycardia, Rishniw et al. compared atropine 0.02 mg/kg given intravenously, intramuscularly, or subcutaneously [15]. Transient atrioventricular block occurred when atrial rate increased before ventricular rate.

Our prior human-anesthesia paper proposed a pharmacophysiological explanation for why antimuscarinic-associated sinus-rate acceleration can appear threshold-like at the bedside [1]. In brief, the observed sinus-rate response reflects the interaction among muscarinic inhibition at the sinoatrial node, sympathetic background, intrinsic sinus-node behavior, the effects of anesthetic drugs, and the current reflex context. Across part of the exposure range, additional muscarinic blockade may produce little visible chronotropic change; over another range, a relatively small further reduction in restraint may be followed by a steep increase in sinus rate. This remains a mechanistic hypothesis for dogs; the cited single-dose observations did not test the proposed relation during incremental antimuscarinic dosing.

If that steep sinus-rate response is expressed after vasopressor exposure has already increased vascular tone, the rate increase may contribute to an abrupt or excessive MAP rise on that high-afterload background.

## 6. Scope and Limits of the Concept

### 6.1. Testable Predictions and Evaluation

The concept concerns the contribution of sinoatrial muscarinic restraint to the vasopressor response, rather than all determinants of MAP. The proposed benefit is more graded MAP control: reaching and maintaining a specified MAP range with fewer abrupt or excessive pressure increases during dose adjustment. Evidence of reduced reflex sinus slowing alone does not establish that this benefit has been achieved.

One possible test is a randomized comparison of antimuscarinic pretreatment and control during anesthesia, with continuous arterial pressure and electrocardiographic recording and the same predefined MAP-guided dosing algorithm for a specified vasopressor. The target MAP range, observation period, dose-adjustment rules, and rescue criteria should be specified in advance for both groups. Recording should begin before pretreatment to include any initial pressure or rhythm disturbance. Isolating the contribution of timing would additionally require a prospectively defined later-antimuscarinic comparison.

A candidate primary endpoint is the time-integrated MAP excess above the predefined upper limit (mmHg·min), assessed alongside time to target and the percentage of observation time within the target range. Dose-to-dose MAP increments, maximum overshoot, hypotension burden, and the number of adjustments provide complementary information. A reduction in overshoot must be interpreted alongside any delay in target attainment or increase in hypotension burden. If reflex sinus slowing is reduced but the prespecified MAP-control endpoint is not improved, the result would not support the proposed titration benefit.

When arrhythmia or atrioventricular conduction disturbance causes monitored HR to differ from sinus rate, changes in HR cannot be treated directly as sinus-rate responses. Changes in anesthetic drug administration or surgical stimulation during dose adjustment need to be distinguished from the response to the vasopressor itself.

Even if the decoupled pattern is observed, MAP and HR alone do not show whether cardiac output, stroke volume, organ blood flow, or tissue perfusion improved. Cardiac output or index, stroke volume, and appropriate perfusion indices can be measured in the same experiment as distinct mechanistic, safety, and secondary outcomes.

### 6.2. Pharmacological Implementation and Safety

Cardiac vagal decoupling identifies a cardiac physiological target, not a cardiac-selective drug action. The relevant sequence is the reduction of sinoatrial muscarinic restraint before vasopressor escalation; its timing within the anesthetic plan can vary. In dogs with morphine- and fentanyl-induced bradycardia, Rishniw et al. found that transient atrioventricular block developed and resolved earlier after intravenous atropine (0.02 mg/kg) than after intramuscular or subcutaneous administration [15]. In conscious dogs, Hendrix and Robinson found that HR after intravenous atropine (30 or 60 μg/kg) was significantly higher than with saline at the first measurement (1 min) and remained higher through 30 and 60 min, respectively [16]. These observations describe the time courses of conduction and chronotropic responses, not the degree or duration of cardiac vagal decoupling. Future studies should therefore compare antimuscarinic drugs, doses, routes, and regimens, including single or repeated boluses and continuous infusion. These comparisons should assess how the degree and duration of reduced muscarinic restraint affect MAP control and the accompanying cardiac and extracardiac responses throughout the titration period.

The hypertension and tachyarrhythmias reported with medetomidine and atropine [13] warrant explicit safety endpoints in prospective titration studies. Lemke et al. found that intravenous atropine (0.1 mg/kg) or glycopyrrolate (0.025 mg/kg) given after xylazine in six isoflurane-anesthetized dogs increased cardiac index and rate–pressure product from post-xylazine values, while stroke volume index remained below the pre-xylazine baseline [17]. Rate–pressure product is an indirect index related to myocardial oxygen demand; its increase did not demonstrate myocardial ischemia or injury in that experiment. Tachycardia may increase myocardial oxygen demand and shorten diastolic filling time, concerns that merit particular assessment in dogs with cardiovascular disease [17].

In a crossover study completed with six conscious dogs, Hendrix and Robinson found that intravenous atropine suppressed small-intestinal motility at doses that also increased HR [16]. This demonstrates an extracardiac action relevant to safety, but the study measured intestinal contractile activity rather than the incidence of perioperative ileus.

## 7. Conclusions

Cardiac vagal decoupling in canine anesthesia is proposed here as a physiological concept for graded vasopressor titration. Reducing sinoatrial muscarinic restraint may lessen reflex sinus slowing during pressor administration. The central hypothesis is that, when subsequent vasopressor doses are adjusted according to the observed MAP response, this reduction may permit more graded pressure control.

This titration hypothesis has not yet been directly tested. Even if MAP control improves, effects on cardiac output, tissue perfusion, and clinical outcomes require separate evaluation.

## Data Availability

No new data were created or analyzed in this study. Data sharing is not applicable to this article.

## Abbreviations

The following abbreviations are used in this manuscript:

HR	Heart rate
MAP	Mean arterial pressure

## References

[1] Nagahama S. (2026). Cardiac Vagal Decoupling: A Conceptual Basis for Reflex-Independent Hemodynamic Management under General Anesthesia. Front. Anesthesiol..

[2] Little C.J., Julu P.O., Hansen S., Reid S.W. (1999). Real-Time Measurement of Cardiac Vagal Tone in Conscious Dogs. Am. J. Physiol..

[3] O’Leary D.S., Rossi N.F., Churchill P.C. (1997). Substantial Cardiac Parasympathetic Activity Exists during Heavy Dynamic Exercise in Dogs. Am. J. Physiol..

[4] Schuessler R.B., Canavan T.E., Boineau J.P., Cox J.L. (1988). Baroreflex Modulation of Heart Rate and Initiation of Atrial Activation in Dogs. Am. J. Physiol..

[5] Tanaka M., Nishikawa T. (2003). Hemodilution Does Not Alter Arterial Baroreflex Control of Heart Rate in Anesthetized Dogs. Anesth. Analg..

[6] Frankel R.A., Metting P.J., Britton S.L. (1993). Evaluation of Spontaneous Baroreflex Sensitivity in Conscious Dogs. J. Physiol..

[7] Fahim M., Arndt J.O. (1990). Left Atrial Receptors in Arterial Baroreflex Control of Heart Rate. Jpn. J. Physiol..

[8] Seagard J.L., Elegbe E.O., Hopp F.A., Bosnjak Z.J., von Colditz J.H., Kalbfleisch J.H., Kampine J.P. (1983). Effects of Isoflurane on the Baroreceptor Reflex. Anesthesiology.

[9] Bagshaw R.J., Cox R.H. (1988). Baroreceptor Control of Central and Regional Hemodynamics with Isoflurane in the Dog. Acta Anaesthesiol. Scand..

[10] Szilagyi J.E. (1987). Opioid Modulation of Baroreceptor Reflex Sensitivity in Dogs. Am. J. Physiol..

[11] Seki D., Goya S., Teshima K., Yamaya Y. (2025). Effects of the Prophylactic Administration of Atropine on the Ephedrine-Induced Cardiac Baroreceptor Reflex in Anesthetized Dogs. J. Vet. Emerg. Crit. Care.

[12] Seki D., Goya S., Teshima K., Yamaya Y. (2025). Emergency Administration of Ephedrine for General Anesthesia-Induced Hypotension Requires Preemptive Atropine in Dogs but Not Cats. J. Am. Vet. Med. Assoc..

[13] Alibhai H.I., Clarke K.W., Lee Y.H., Thompson J. (1996). Cardiopulmonary Effects of Combinations of Medetomidine Hydrochloride and Atropine Sulphate in Dogs. Vet. Rec..

[14] Becker D.E. (2012). Basic and Clinical Pharmacology of Autonomic Drugs. Anesth. Prog..

[15] Rishniw M., Tobias A.H., Slinker B.K. (1996). Characterization of Chronotropic and Dysrhythmogenic Effects of Atropine in Dogs with Bradycardia. Am. J. Vet. Res..

[16] Hendrix P.K., Robinson E.P. (1997). Effects of a Selective and a Nonselective Muscarinic Cholinergic Antagonist on Heart Rate and Intestinal Motility in Dogs. J. Vet. Pharmacol. Ther..

[17] Lemke K.A., Tranquilli W.J., Thurmon J.C., Benson G.J., Olson W.A. (1993). Hemodynamic Effects of Atropine and Glycopyrrolate in Isoflurane-Xylazine-Anesthetized Dogs. Vet. Surg..

